# Dulaglutide and neurodegeneration biomarkers: REWIND post hoc analysis

**DOI:** 10.1002/alz.71391

**Published:** 2026-04-16

**Authors:** Jonathan M. Wilson, Jeffrey L. Dage, Hui‐Rong Qian, Courtney L. Irelan, Hannah S. Crowder, Kevin L. Duffin, Mark Mintun, Dawn A. Brooks, Hertzel C. Gerstein, M. Angelyn Bethel

**Affiliations:** ^1^ Eli Lilly and Company, Lilly Corporate Center Indianapolis Indiana USA; ^2^ Stark Neurosciences Research Institute Indiana University School of Medicine Indianapolis Indiana USA; ^3^ Population Health Research Institute Hamilton Ontario Canada

**Keywords:** Alzheimer's disease, cognitive impairment, dulaglutide, glial fibrillary acidic protein, phosphorylated tau217, plasma neurofilament light chain

## Abstract

**INTRODUCTION:**

This post hoc analysis of the Researching Cardiovascular Events with a Weekly Incretin in Diabetes (REWIND) trial examined associations between dulaglutide, Alzheimer's disease and related dementia (ADRD) biomarkers, and substantive cognitive impairment (SCI).

**METHODS:**

Participants (dulaglutide, *n* = 3741; placebo, *n* = 3627) completed cognitive tests and provided blood samples at baseline and 2 years, with additional cognitive tests at 5‐year and final visits. Analysis of covariance and Cox models tested associations between dulaglutide, SCI, and plasma neurofilament light chain (NfL), phosphorylated tau217 (p‐tau217), and glial fibrillary acidic protein (GFAP) biomarkers.

**RESULTS:**

Dulaglutide‐associated changes in biomarkers or SCI were nonsignificant overall. Participants with NfL ≥56 pg/mL had a dulaglutide‐associated 2‐year reduction in NfL (−27.4% vs −14.2%; *p *= 0.003). Participants with p‐tau217 ≥25 pg/mL had a dulaglutide‐associated SCI reduction (hazard ratio = 0.78; *p *= 0.0064).

**DISCUSSION:**

Dulaglutide reduced NfL and SCI in participants with select elevated plasma ADRD biomarkers, with limited 2‐year effects on p‐tau217 and GFAP. Future studies with longer follow‐up should explore incretin‐related cognitive changes.

**CLINICAL TRIAL REGISTRATION INFORMATION:**

NCT01394952, registered July 15, 2011.

## Highlights

- Treatment‐related 5‐year reduction in subjective cognitive impairment (SCI) occurred in a sub‐group of participants.
- The 22% reduction in SCI was significant in those with baseline phosphorylated tau217 (p‐tau17) ≥0.25 mg/dL.
- Over 2 years, dulaglutide reduced plasma NfL in those with baseline NfL ≥56 pg.ml.

## 1 BACKGROUND

Type 2 diabetes (T2D) is an independent risk factor for cognitive impairment and Alzheimer's disease (AD).^1^, ^2^ Although the reasons for cognitive impairment in people with diabetes are likely multifactorial, a large body of evidence implicates cardiovascular disease in its pathogenesis.^3^ Plasma biomarkers of AD and related dementias (ADRD) may provide a better understanding of the mechanisms linking T2D to neurodegeneration and cognitive impairment.

The Researching Cardiovascular Events with a Weekly Incretin in Diabetes (REWIND) cardiovascular outcomes trial reported that a once‐weekly subcutaneous injection of dulaglutide 1.5 mg reduced the hazard of major adverse cardiovascular events—a composite outcome including cardiovascular‐related death, myocardial infarction, or stroke—by 12% over a median follow‐up of 5.4 years in 9901 adults with T2D with varying levels of cardiovascular risk.^4^ Furthermore, in an exploratory analysis of 8828 participants from the REWIND trial with baseline and follow‐up scores from the Montreal Cognitive Assessment (MoCA) or Digit Symbol Substitution Test (DSST), dulaglutide was associated with a 14% reduced risk of country‐standardized substantive cognitive impairment (SCI), defined as the first occurrence of a follow‐up score on the MoCA or DSST with a standard deviation (SD) of 1.5 or more below the mean baseline country‐standardized score.^5^

The plasma biomarkers neurofilament light chain (NfL), phosphorylated tau217 (p‐tau217), and glial fibrillary acidic protein (GFAP) have been linked to AD and cognitive impairment.^6^, ^7^, ^8^, ^9^ NfL originates in central and peripheral compartments and is associated with neurodegeneration such as neuropathies, multiple sclerosis, AD, and vascular dementia.^5^, ^10^ P‐tau217 originates in the central compartment^11^ and is associated with preclinical and clinical AD pathology.^12^, ^13^, ^14^, ^15^, ^16^, ^17^ The levels of p‐tau217 are known to predict AD and cognitive decline.^6^, ^18^, ^19^, ^20^, ^21^ GFAP, an indicator of reactive astrogliosis, is associated with preclinical AD^22^, ^23^, ^24^ and a high amyloid beta (Aβ) signal on positron emission tomography scans.^25^ Although all three biomarkers have been explored in populations with various chronic conditions,^15^, ^19^, ^26^, ^27^ to the best of our knowledge, they have not been investigated in a large cardiovascular outcomes study among adults with T2D.

In the present post hoc exploratory analysis, we examined baseline NfL, p‐tau217, and GFAP biomarker levels and their association with each other, with baseline cognitive MoCA and DSST scores, and with 5‐year incident SCI for the first time in the context of a randomized controlled trial (REWIND). We also explored the effect of dulaglutide treatment versus placebo on changes in these biomarker levels over 2 years and further investigated treatment‐associated effects on SCI over 5 years by biomarker subgroups, categorized by baseline levels.

## 2 METHODS

### 2.1 Study design and participants

**FIGURE 1 alz71391-fig-0001:**
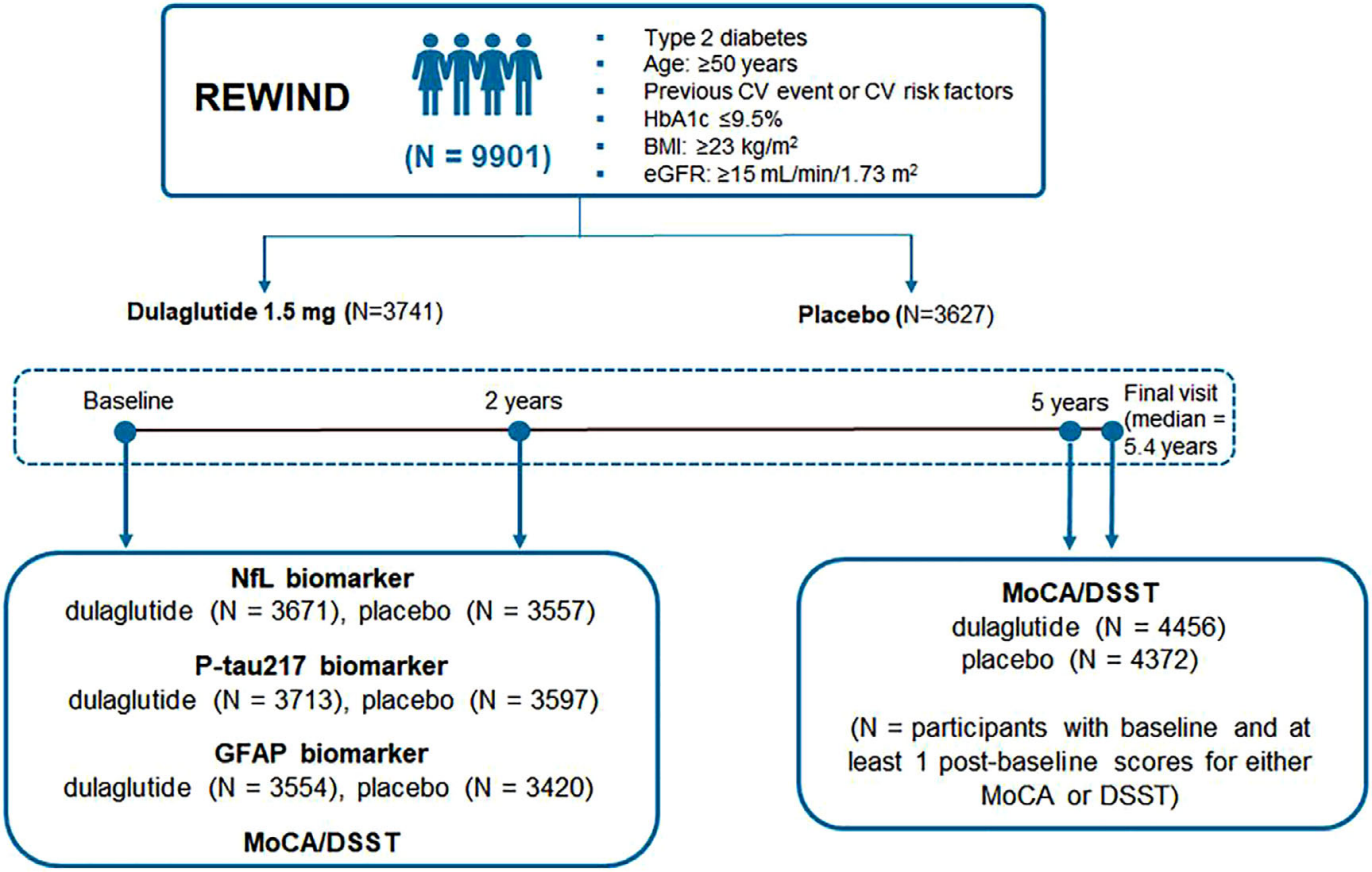
Study design, participant recruitment, and sample collection strategy for participants providing data for the post hoc analysis of cognitive biomarkers and cognitive testing. BMI, body mass index; CV, cardiovascular; DSST, Digit Symbol Substitution Test; eGFR, estimated glomerular filtration rate; GFAP, glial fibrillary acidic protein; HbA1c, glycated hemoglobin; MoCA, Montreal Cognitive Assessment; NfL, neurofilament light chain; p‐tau217, phosphorylated tau217.

The study design and key inclusion criteria of the REWIND trial (ClinicalTrials.gov identifier: NCT01394952; registered on July 15, 2011) are shown in Figure 1, with details published previously.^4^, ^28^ From August 2011 through August 2013, this multicenter, international (371 sites in 24 countries) clinical cardiovascular outcomes trial enrolled men and women at least 50 years of age who had T2D and, a previous cardiovascular event or cardiovascular risk factors, glycated hemoglobin ≤9.5% (≤80 mmol/mol), and body mass index ≥23 kg/m^2^, and were taking up to two oral glucose‐lowering drugs, with or without basal insulin, at stable doses for at least 3 months. Individuals were ineligible for trial participation if they had a coronary or cerebrovascular event within the previous 2 months; had an estimated glomerular filtration rate < 15 mL/min/1.73 m^2^ or were undergoing renal dialysis; had severe hypoglycemia within the past year, cancer within the past 5 years, or had a history of pancreatitis, bariatric surgery, or known abnormal gastric emptying; or if they previously participated in any study investigating dulaglutide. Over the median follow‐up of 5.4 years in REWIND, 8828 participants (dulaglutide, *n* = 4456; placebo, *n* = 4372) had a baseline score and at least one follow‐up score for MoCA or DSST cognitive testing. Of these 8828 participants, the analyses reported herein were conducted on the 7368 participants (83.5%) with available biomarker data at both baseline and Year 2 visits (dulaglutide, *n* = 3741; placebo, *n* = 3627).

The REWIND trial was conducted in accordance with the ethical standards as laid down in the 1964 Declaration of Helsinki and its later amendments or comparable ethical standards. Research ethics boards at each study site approved the REWIND trial protocol, and all participants provided written informed consent before any trial‐related procedures. The international setting of this study facilitated the inclusion of underrepresented groups.

### 2.2 Country standardization of MoCA and DSST scores and SCI

The cognitive status of participants was assessed by two validated measures of cognitive function at the study's baseline, 2‐year, 5‐year, and end‐of‐study visits: the MoCA and the DSST. Raw MoCA and DSST scores were converted to country‐specific standardized scores for each participant to account for evidence that the normal range of cognitive test scores might differ by country due to regional or cultural differences.^29^, ^30^ This conversion was done by first calculating the baseline mean and SD of the MoCA and DSST score for every country. These values were then used to calculate a standardized MoCA and DSST score for every participant at each timepoint by subtracting the country‐specific baseline mean raw score from the participant's raw score at that timepoint and then dividing the difference by the country‐specific baseline SD. Thus, the mean (SD) baseline standardized score of all participants for both the MoCA and the DSST was 0 (1) within every country.

An SCI event was considered to be the first occurrence of a follow‐up score on the MoCA or DSST with an SD of at least 1.5 below the mean baseline country‐standardized score.^5^ As previously published, the 4456 participants from the REWIND study who were assigned to dulaglutide had a 14% reduced hazard of an SCI event as compared to the 4372 participants assigned to placebo (hazard ratio [HR] = 0.86; 95% confidence interval [CI]: 0.79–0.95; *p *= 0.0018).^5^

### 2.3 Biomarker analysis

All three ADRD biomarkers were measured without knowledge of any clinical data or dulaglutide treatment assignments. Fasting plasma samples were collected from participants at baseline and 2 years and stored at −80°C in dipotassium ethylenediaminetetraacetic acid (K_2_EDTA) tubes, and then centrifuged to collect plasma, which was then stored in a separate aliquoted tube until analysis. Baseline levels of NfL, p‐tau217, and GFAP were assayed in 3671, 3713, and 3554 participants assigned to dulaglutide, respectively, and in 3557, 3597, and 3420 participants assigned to placebo, respectively. NfL concentrations were measured in a twofold dilution of EDTA plasma using the Ella platform (ProteinSimple; Bio‐Techne, Minneapolis, MN, USA).^31^ Levels of p‐tau217 were quantified in EDTA plasma with a twofold dilution using the Meso Scale Discovery platform (MSD, Rockville, MD, USA). GFAP levels were measured in EDTA plasma at a 1:8 dilution using the Quanterix platform (Quanterix, Billerica, MA, USA).

**RESEARCH IN CONTEXT**

1. **Systematic review**: We reviewed the literature per traditional sources (e.g., PubMed). Few articles have been published that explore associations between glucagon‐like peptide‐1 receptor agonists, cognitive outcomes, and plasma levels of neurofilament light chain (NfL), phosphorylated tau217 (p‐tau217), and glial fibrillary acidic protein (GFAP), and none were clinical trials.
2. **Interpretation**: In participants with p‐tau217 levels correlating with Alzheimer's disease (AD) pathology (≥0.25 pg/mL), treatment with dulaglutide was associated with a 22% reduced risk of substantive cognitive impairment. In participants with NfL levels highly correlated with neurodegeneration (≥56 pg/mL), treatment with dulaglutide was associated with a significant 2‐year reduction in NfL.
3. **Future directions**: Additional investigations are needed to study the effects of glucagon‐like peptide‐1 receptor agonists on biomarkers of cognitive decline. Future studies, with better‐defined subpopulations of participants and their responses to incretins, are needed to help better understand the effects of incretins on cognitive decline and neurodegeneration.

#### 2.3.1 Biomarker subcategory definitions

Cut‐points for each biomarker were chosen based on prior literature reporting on NfL, GFAP, and p‐tau217 levels. GFAP levels were classified into low (<121 pg/mL), mid‐level (≥121–≤180 pg/mL), and high (>180 pg/mL) tertiles to create GFAP subgroups.^32^ The cut‐point for NfL was ≥56 pg/mL, corresponding to the 90th percentile—a level with more than 80% sensitivity and specificity for neurodegenerative and cerebrovascular disease.^31^, ^33^ The cut‐point for p‐tau217 was ≥0.25 pg/mL, a level previously associated with AD pathology.^15^, ^34^, [Fig alz71391-fig-0001]

### 2.4 Statistical analysis

Baseline demographic data were summarized using means and SDs or median and interquartile ranges (IQRs) for continuous variables and counts and percentages for categorical variables. Pearson correlations, both unadjusted and adjusted by controlling for age, sex, and age‐by‐sex interaction, were calculated to evaluate the relationship between baseline biomarker levels and baseline country‐standardized cognitive test scores. Biomarker data were analyzed on a log scale and then represented as concentrations by back transformation to a linear scale. The *p*‐values were not adjusted for multiplicity testing given the exploratory nature of these analyses.

Primary endpoint: Analysis of covariance models were used to test if the effect of dulaglutide on the change in biomarker levels over 2 years varied with each baseline biomarker level. These models assessed 2‐year changes in biomarker levels as the outcome and included baseline biomarker level, dulaglutide or placebo treatment assignment, and a baseline biomarker‐by‐treatment interaction term. Baseline biomarkers were treated as either a continuous or categorical variable in subgroups. Analyses with similar models, restricted to each subgroup as defined by the previously described cut‐points, were also conducted to test the effects of dulaglutide treatment. As these models were restricted to subgroups, the biomarker‐by‐treatment interaction term was excluded.

Secondary endpoint: To estimate the effect of dulaglutide treatment on SCI, a Cox proportional hazards model was used for time from randomization to an SCI event, with standardized baseline MoCA and DSST scores as covariates and treatment as the model term. This was applied to the overall population and to biomarker subgroups defined by the previously described cut‐points. A similar model, with additional model terms of baseline biomarker subgroup and biomarker‐by‐treatment interaction, was also applied to test if baseline biomarker levels impact the effect of treatment on SCI.

## 3 RESULTS

### 3.1 Characteristics, biomarker levels, and cognitive test scores of participants at baseline

#### 3.1.1 Demographics and characteristics

Baseline demographics and characteristics of participants were comparable between treatment groups (Table 1). Participants had a median (IQR) age of 65.5 (61.4–70.0) years and a mean (SD) diabetes duration of 10.2 (7.0) years. Almost half of the participants were female (48%), and about one‐third (31%) had a history of cardiovascular disease. Median (IQR) biomarker levels at baseline were 26.5 (19.8–37.5) pg/mL for NfL, 147 (107–201) pg/mL for GFAP, and 0.20 (0.16–0.26) pg/mL for p‐tau 217. The p‐tau217 level was at or above the AD cut‐point of 0.25 pg/mL in 29% of participants.

**TABLE 1:** Demographics and baseline characteristics of participants in the post hoc biomarker analysis.

	Overall (*N* = 7368)	Dulaglutide (*N* = 3741)	Placebo (*N* = 3627)
Age, years	65.5 (61.4–70.0)	65.4 (61.5–70.1)	65.6 (61.4–70.0)
Female sex	3514 (48%)	1790 (48%)	1724 (48%)
White ethnic origin	5644 (77%)	2884 (77%)	2760 (76%)
Education of ≤12 years	4605 (63%)	2338 (63%)	2267 (63%)
Current tobacco use	1035 (14%)	527 (14%)	508 (14%)
Diabetes duration, years	10.2 ± 7.0	10.1 ± 7.0	10.3 ± 6.9
Cardiovascular disease^a^	2283 (31%)	1151 (31%)	1132 (31%)
Stroke or TIA	671 (9%)	342 (9%)	329 (9%)
Atrial fibrillation	471 (6%)	240 (6%)	231 (6%)
Heart failure	677 (9%)	335 (9%)	342 (9%)
Hypertension	6860 (93%)	3482 (93%)	3378 (93%)
HbA1c, %	7.3 ± 1.1	7.3 ± 1.1	7.3 ± 1.1
Body mass index, kg/m^2^	32.3 ± 5.7	32.4 ± 5.7	32.3 ± 5.7
eGFR, mL/min/1.73 m^2^	77.9 ± 23.7	78.5 ± 23.6	77.3 ± 23.8
Albuminuria^b^	2491 (34%)	1249 (33%)	1242 (34%)
Systolic blood pressure, mm Hg	138.0 ± 16.7	137.0 ± 16.4	138.0 ± 17.0
Diastolic blood pressure, mm Hg	78.8 ± 9.8	78.8 ± 9.7	78.8 ± 9.9
LDL cholesterol, mmol/L	2.6 ± 1.0	2.6 ± 1.0	2.6 ± 1.0
SCI events^c^	1515 (21%)	735 (20%)	780 (22%)
DSST score	36 (25–48)	36 (25–49)	36 (25–48)
MoCA score	25 (22–28)	25 (23–28)	25 (22–28)
NfL, pg/mL	26.5 (19.8–37.5)	26.1 (19.9–37.4)	26.8 (19.7–37.7)
p‐tau217, pg/mL	0.20 (0.16–0.26)	0.20 (0.16–0.26)	0.20 (0.16–0.26)
p‐tau217 ≥0.25 pg/mL	2105 (29%)	1063 (28%)	1042 (29%)
GFAP, pg/mL	147 (107–201)	148.1 (107.9–203.9)	145.9 (107.4–199.2)

*Note*: Data are shown as n (%), mean ± SD, or median (interquartile range).

Abbreviations: DSST, Digit Symbol Substitution Test; eGFR, estimated glomerular filtration rate; GFAP, glial fibrillary acidic protein; HbA1c, glycated hemoglobin; Hg, mercury; kg, kilograms; L, liters; LDL, low‐density lipoprotein; m, meters; mg, milligrams; min, minutes; mL, milliliters; mm, millimeters; mmol, millimoles; MoCA, Montreal Cognitive Assessment; N, number; NfL, neurofilament light chain; p‐tau217, phosphorylated tau217; pg, picograms; SCI, substantive cognitive impairment; SD, standard deviation; TIA, transient ischemic attack.

^a^ Includes myocardial infarction, ischemic stroke, unstable angina with changes on electrocardiography, myocardial ischemia on imaging or stress test, or coronary, carotid, or peripheral revascularization.

^b^ Urinary albumin‐to‐creatinine ratio ≥3.39 mg/mmol.

^c^ Defined as the first occurrence of a follow‐up score on the MoCA or DSST with an SD of at least 1.5 below the mean baseline country‐specific standardized score.

#### 3.1.2 Biomarkers and cognitive test scores

Baseline NfL, GFAP, and p‐tau217 levels were positively associated with each other before and after adjusting for age, sex, and age‐by‐sex interaction (all *p *< 0.001) (Table 2). After adjusting for age, sex, and age‐by‐sex interaction, baseline NfL was negatively associated with baseline country‐specific standardized MoCA and DSST scores (both *p *< 0.001). No significant associations were found between baseline country‐specific standardized MoCA or DSST scores and p‐tau217 or GFAP levels after adjustment (Table 2).

**TABLE 2:** Unadjusted and adjusted correlations between each baseline plasma biomarker and country‐specific standardized cognitive test scores and other biomarkers.

Biomarker	Model	MoCA score	DSST score	p‐tau217	GFAP
NfL	Unadjusted	−0.082^***^	−0.108^***^	0.131^***^	0.248^***^
	Adjusted	−0.041^***^	−0.056^***^	0.104^***^	0.168^***^
p‐tau217	Unadjusted	−0.028^**^	−0.042^***^	—	0.201^***^
	Adjusted	−0.008 ^ns^	−0.015 ^ns^	—	0.169^***^
GFAP	Unadjusted	−0.107^***^	−0.121^***^	—	—
	Adjusted	−0.020 ^ns^	−0.012 ^ns^	—	—

*Note*: Adjusted models include age, sex, and age‐by‐sex interaction term.

Abbreviations: DSST, Digit Symbol Substitution Test; GFAP, glial fibrillary acidic protein; MoCA, Montreal Cognitive Assessment; NfL, neurofilament light chain; p‐tau 217, phosphorylated tau217.

*** *p *< 0.001.

** *p* < 0.05; non‐significant (ns) *p *> 0.05.

### 3.2 Treatment‐related changes in biomarker levels at 2 years

#### 3.2.1 NfL

**FIGURE 2 alz71391-fig-0002:**
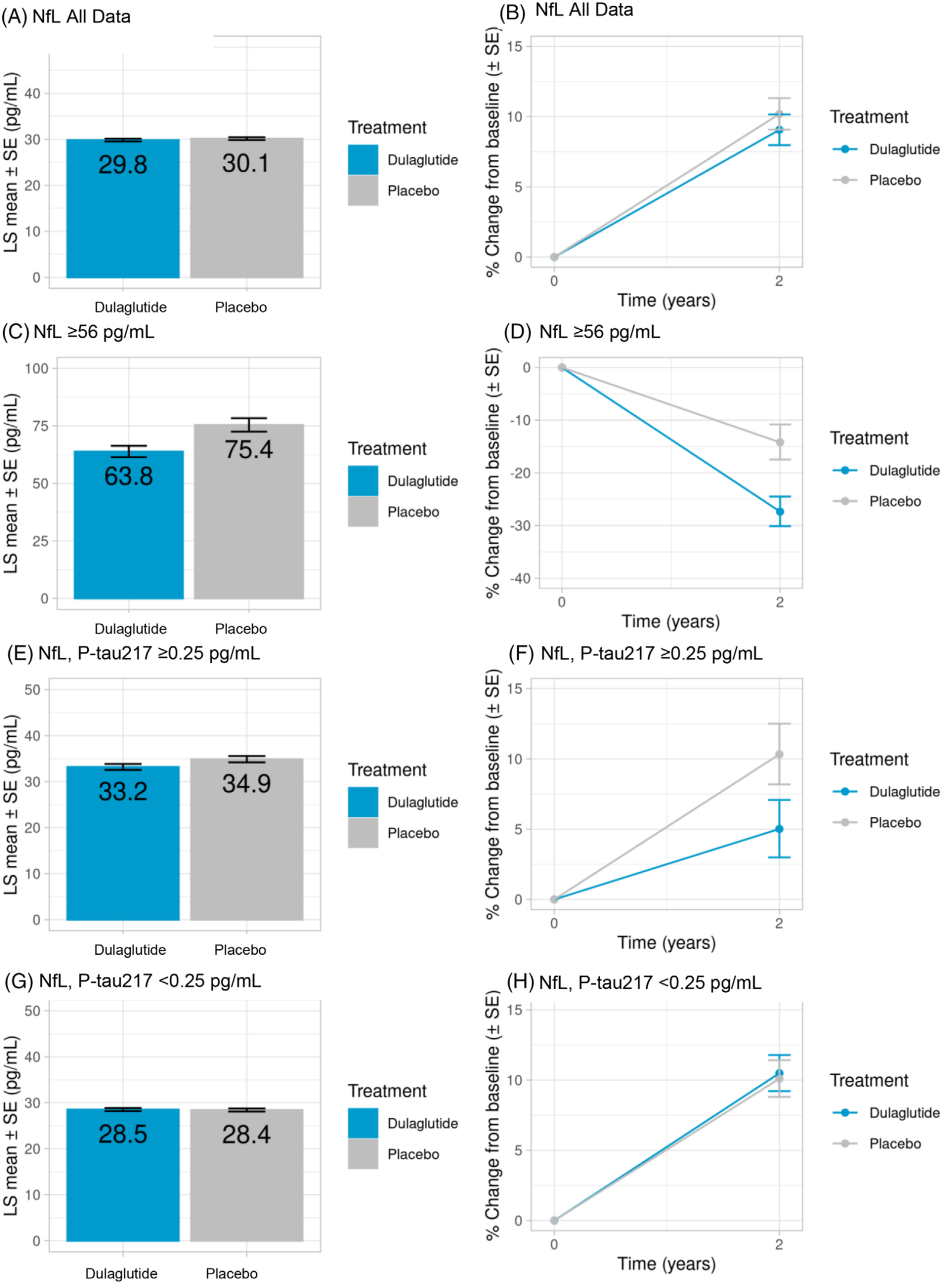
NfL levels at 2 years in the overall population (NfL all data; A and B) and in NfL subgroups (C–H). Data are shown as LS mean (SE) for all graphs on the left and as percent change from baseline (SE) for all graphs on the right. Interaction *p*‐values: NfL all data, *p *= 0.47; NfL ≥56 pg/mL (“High10”), *p *= 0.003; NfL with p‐tau217 ≥0.25 pg/mL, *p *= 0.07; NfL with p‐tau217 < 0.25 pg/dL, *p *= 0.83. LS, least squares; NfL, neurofilament light chain; p‐tau‐217, phosphorylated tau217; SE, standard error.

NfL concentrations were not significantly changed at 2 years with dulaglutide treatment compared with placebo in the overall trial population, as represented by the baseline‐adjusted plasma NfL level (least‐squares [LS] mean [standard error (SE)]: 29.8 ± 0.03pg/mL vs 30.1 ± 0.03 pg/mL, respectively) and the percent increase from baseline (9.1% vs 10.2%, respectively; *p *= 0.47) at 2 years (Table 3; Figure 2A,B; Table S1). However, there was a statistically significant interaction between the treatment‐related 2‐year change and baseline NfL concentration, treating it as a continuous numeric variable or by dichotomized subgroups (*p *< 0.001) (Table 3). As a result, categories of baseline NfL levels were further analyzed.

**TABLE 3:** Changes in plasma biomarkers by treatment assignment (dulaglutide or placebo) from baseline to Year 2, for overall study population and for subgroups defined by baseline biomarker levels.

Biomarker	Subgroup	Percent difference (dulaglutide vs placebo)	SE	*p‐*value (dulaglutide vs placebo)	Subgroup‐by‐treatment interaction *p‐*value	Biomarker‐by‐treatment interaction *p‐*value
NfL	Overall	−1.04	0.01	0.47	0.0003	<0.0001
	<56 pg/mL	0.61	0.01	0.68	NA	NA
	≥56 pg/mL	−18.0	0.98	0.003		
p‐tau217	Overall	0.97	0.01	0.29	0.78	0.85
	<0.25 pg/mL	0.81	0.01	0.43	NA	NA
	≥0.25 pg/mL	1.38	0.03	0.46		
GFAP	Overall	1.65	0.02	0.07	0.87	0.95
	<121 pg/mL	1.72	0.03	0.31	NA	NA
	≥121 to ≤180 pg/mL	2.14	0.03	0.09		
	>180 pg/mL	1.01	0.02	0.52		

Abbreviations: GFAP, glial fibrillary acidic protein; mL, milliliters; NA, not applicable; NfL, neurofilament light chain; p‐tau 217, phosphorylated tau217; pg, picograms; SE, standard error.

Treatment with dulaglutide was associated with a significant decline in NfL levels in the NfL ≥56 pg/mL subgroup versus placebo (*p *= 0.003). Baseline‐adjusted LS mean (SE) NfL levels for dulaglutide versus placebo, respectively, were 63.8 ± 2.5 pg/mL versus 75.4 ± 3.0 pg/mL, with a percent change from baseline of −27.4% versus −14.2% (both *P *< 0.001) (Table 3; Figure 2C,D; Table S1). In addition, there was a trend toward lower NfL levels over 2 years with dulaglutide versus placebo among participants in the NfL ≥56 pg/mL subgroup with a p‐tau217 level ≥0.25 pg/mL (*p *= 0.07), but not in those with NfL ≥56 pg/mL with a p‐tau217 level < 0.25 pg/mL (Figure 2E–H; Table S1).

#### 3.2.2 P‐tau217

**FIGURE 3 alz71391-fig-0003:**
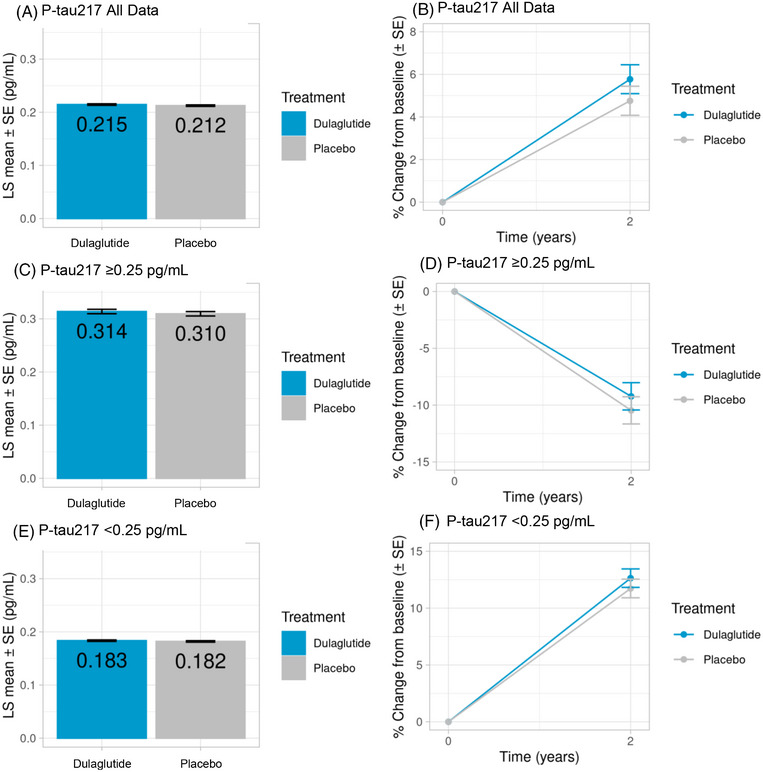
Levels of p‐tau217 at 2 years in the overall population (A and B) and in p‐tau217 subgroups (C–F). Data are shown as LS mean (SE) for all graphs on the left and as percent change from baseline (SE) for all graphs on the right. Interaction *p*‐values > 0.05 for overall (p‐tau217 all data) and all p‐tau217 subgroups. LS, least squares; p‐tau217, phosphorylated tau217; SE, standard error.

The 2‐year adjusted levels of plasma p‐tau217 in the dulaglutide versus placebo groups (LS mean [SE]: 0.215 ± 0.001 pg/mL vs 0.213 ± 0.001 pg/mL, respectively) and the percent increase from baseline (5.8% vs 4.8%) were not significantly different (*p *= 0.29) (Figure 3A,B; Table S1). Although there was no significant interaction between dulaglutide treatment and baseline p‐tau217 level (*P *= 0.85) (Table 3), treatment‐associated effects on subgroups were further examined a priori, yielding no significant differences (Figure 3C–F; Table S1).

#### 3.2.3 GFAP

**FIGURE 4 alz71391-fig-0004:**
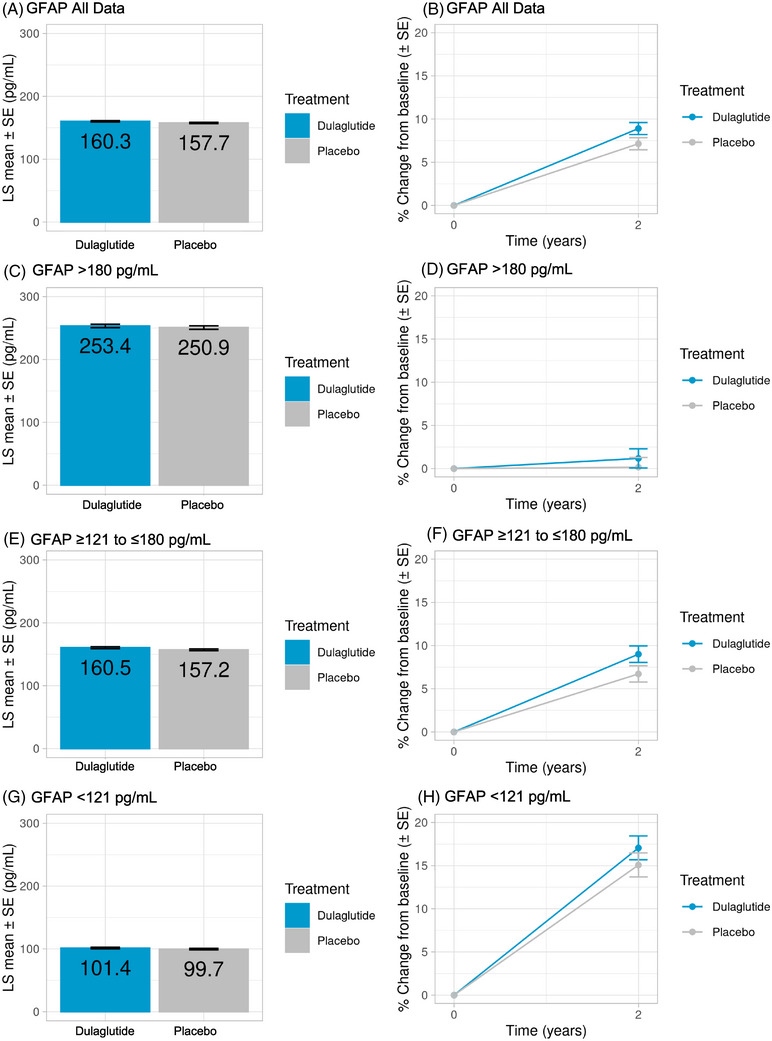
GFAP levels at 2 years in the overall population (all data; A and B) and in GFAP tertile subgroups (C–H). Data are shown as LS mean (SE) for all graphs on the left and as percent change from baseline (SE) for all graphs on the right. Interaction *p*‐values > 0.05 for overall GFAP (GFAP All Data) and all GFAP subgroups. BL, baseline; GFAP, glial fibrillary acidic protein; LS, least squares; SE, standard error.

At 2 years, there was no statistically significant treatment difference seen with dulaglutide versus placebo for the baseline‐adjusted plasma GFAP level (LS mean [SE]: 160.3 ± 1.0 pg/mL vs 157.7 ± 1.0 pg/mL, respectively; *P *= 0.07) or the percent change from baseline (8.9% vs 7.1%, respectively) (Figure 4A,B; Table S1). There was also no significant interaction observed between dulaglutide treatment and baseline GFAP level (*p *= 0.95) (Table 3).[Fig alz71391-fig-0002], [Fig alz71391-fig-0003], [Fig alz71391-fig-0004]

A priori, treatment‐associated 2‐year changes in GFAP were studied further by tertiles of baseline GFAP levels. No significant changes were observed with dulaglutide treatment versus placebo in any of the GFAP subgroups at 2 years, as represented by the baseline‐adjusted LS mean (SE) GFAP levels in the high (253.43 vs 250.88 pg/mL; *P *= 0.52), mid‐level (160.53 vs 157.17 pg/mL; *P *= 0.09), and low subgroups (101.37 vs 99.65 pg/mL; *P *= 0.31) (Figure 4C–H; Table S1).

### 3.3 Treatment‐related changes in SCI at 5 years

**FIGURE 5:**
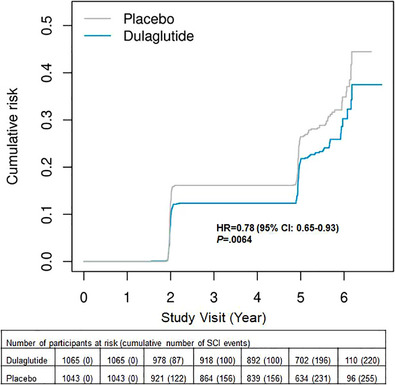
Cumulative incidence of SCI events in REWIND participants with baseline p‐tau217 ≥0.25 pg/mL. CI, confidence interval; HR, hazard ratio; mL, milliliters; pg, picgrams; p‐tau217, phosphorylated tau217; REWIND, Researching Cardiovascular Events with a Weekly Incretin in Diabetes; SCI, substantive cognitive impairment.

Participants assigned to dulaglutide with a baseline p‐tau217 level ≥0.25 pg/mL, which correlates to AD pathology, had a significant reduction in SCI as compared to participants assigned to placebo (HR = 0.78; 95% CI: 0.65–0.93; *P *= 0.0064) (Figure 5). This reduced risk of SCI was similar to the results previously reported for the overall trial population (HR = 0.86).^5^ The risk of SCI did not significantly differ by treatment for other biomarker subgroups (data not shown).

## 4 DISCUSSION

This unique study characterized relevant ADRD biomarkers in a large longitudinal trial with associated outcomes and endpoints, providing insights into the effect of dulaglutide treatment on ADRD biomarkers. The three plasma biomarkers included in this study were previously shown to be associated with the progression of neurodegenerative diseases. Incretin‐based therapies, such as glucagon‐like peptide‐1 receptor agonists, have shown promising effects on neurodegenerative pathways,^35^, ^36^, ^37^, ^38^, ^39^ and this study has provided a novel way to examine these effects.

Recent publications reporting on plasma NfL and GFAP concentrations in individuals with T2D show that levels of these biomarkers are comparable to those observed in participants from the REWIND study. Studies by Mielke et al. and others documented that NfL levels in people with diabetes typically range from 15 to 25 pg/mL,^40^, ^41^ whereas GFAP concentrations are often observed within the range of 100–200 pg/mL,^41^, ^42^, ^43^ aligning closely with our findings from the current population studied. In addition, results from a multicenter analysis of individuals with AD or non‐AD dementias by Doecke et al. show that neither NfL nor GFAP levels significantly differ from these biomarker levels in participants from REWIND,^44^ further supporting the consistency of these biomarker profiles across studies. These reports collectively suggest that the levels of NfL and GFAP measured in this cohort should be representative of levels for adults with T2D with or without overt cognitive impairment.

Baseline NfL, GFAP, and p‐tau217 levels were correlated with each other, and these relationships remained statistically significant after adjusting for age and sex. However, of the three biomarkers, only NfL was correlated significantly with cognitive scores, with this association remaining robust after age and sex adjustments. In the NfL ≥56 pg/mL subgroup, dulaglutide treatment was associated with a greater 2‐year reduction in NfL levels than placebo, whereas NfL levels increased over time among the other participants; this rise in NfL over time is expected due to natural aging.^45^ This finding of a dulaglutide‐associated change of NfL in a small subgroup with the highest baseline levels, but no reduction in GFAP or p‐tau217 levels, taken along with lowered cognitive test scores, might indicate that cognitive changes in this population are due to the impact of mixed pathologies, such as cerebrovascular disease. Incretins improve endothelial function, reduce inflammation and oxidative stress, and may prevent microglial activation.^46^, ^47^ We know of only two other large‐scale, randomized, Phase 3 clinical trials examining incretin‐related changes in blood‐based ADRD biomarkers over time: EVOKE and EVOKE+.^48^ Results of these studies are expected to be published later this year and will provide an interesting comparison with our study. Based on our results, we posit that incretins may be particularly effective for addressing vascular neurodegenerative pathology.

The interaction between cognition, tau pathology, and vascular disease has been investigated neuropathologically in a large study across AD centers.^49^ Our results are largely aligned with these findings and provide insights into the extent to which vascular pathology contributes to cognitive decline independently and synergistically with AD pathology. These findings can aid in the understanding of these complex interactions and have implications for treatment and prevention strategies towards both diseases.

In this study, population was unselected for AD pathology, and 29% of participants had a high p‐tau217 level at baseline. Few studies specifically assess p‐tau217 levels in populations with T2D, although p‐tau217 is known to be higher in individuals with a history of chronic kidney disease, myocardial infarction, and stroke.^15^ Routine cases within cardiology clinics have some degree of cognitive impairment, and cognitive decline can be commonly seen in up to one in three patients with cardiovascular disease, depending on cardiac conditions, comorbidities, and age.^50^, ^51^ Although data are limited, a small cross‐sectional study has explored the influence of diabetes and other metabolic comorbidities on p‐tau217 levels. Olvera‐Rojas et al. reported that individuals with T2D exhibited lower plasma p‐tau217 levels compared with participants without diabetes in a cohort focused primarily on AD.^52^ This suggests that although diabetes and related metabolic factors may be linked to changes in p‐tau217, these factors alone do not appear to significantly increase p‐tau217 levels, except in cases of underlying neurodegenerative disease.

Findings from the current analysis also indicate a possible association of dulaglutide with a greater reduction in cognitive impairment among people with p‐tau217 ≥0.25 pg/mL, a range associated with AD. Of note, this subpopulation may overlap with the population that showed NfL‐lowering responses with dulaglutide treatment (Figure 2C,D). Additional quantitative studies among participants with a longer diabetes duration and reduced renal function could be the target of future dedicated studies examining p‐tau217 in well‐characterized T2D cohorts.

Study participants from REWIND with a baseline ‐tau217 level ≥0.25 pg/mL who were assigned to dulaglutide had a 22% reduced risk of SCI, whereas the dulaglutide‐associated reduced risk of SCI in the overall study population was 14%.^5^ This biomarker study of a subpopulation from the REWIND trial has helped clarify the effect of dulaglutide on cognition and deserves further examination with validation cohorts of longer follow‐up duration.

The strengths of this study include the quantification of plasma ADRD biomarkers at both baseline and 2 years from participants in a large randomized trial in an international setting. In addition, widely utilized and validated cognitive assessments (MoCA and DSST) were available for association analyses of these biomarkers and clinical parameters over 5 years of follow‐up. Limitations of this study include the post hoc nature of the analysis and that the biomarker analysis was restricted to a 2‐year follow‐up. Future studies should explore changes in ADRD biomarkers over a longer follow‐up duration in participants treated with dulaglutide. In addition, the power to detect an interaction between treatment and biomarker levels on cognitive impairment was low, so a significant interaction may not have been detected, even if one existed.

In conclusion, 2‐year treatment with dulaglutide appears to modulate plasma NfL levels, with a larger modulation effect observed among participants with higher baseline levels of this ADRD biomarker. Furthermore, the reduced risk of SCI previously demonstrated with dulaglutide might be greater among those with a higher burden of AD pathology. Future studies examining incretin therapies in populations selected for AD or vascular pathologies of neurodegeneration are needed to confirm and extend these observations.

## CONFLICT OF INTEREST STATEMENT

J.M.W., H.R.Q., C.L.I., H.S.C., K.L.D., M.M., D.A.B., and M.A.B. are employees and shareholders of Eli Lilly and Company. J.L.D. is a former employee and minor shareholder of Eli Lilly and Company. J.L.D. is an inventor on patents or patent applications assigned to Eli Lilly and Company relating to the assays, methods, reagents, and/or compositions of matter for p‐tau assays and amyloid‐targeting therapeutics. J.L.D. has served as a consultant or on advisory boards for AbbVie, Alzheimer's Drug Discovery Foundation, ALZpath, Cognito Therapeutics, Dolby Family Ventures, Early Is Good, Eisai, Eli Lilly and Company, Gates Ventures, Genotix Biotechnologies, Karuna Therapeutics, MindImmune Therapeutics, Neurogen Biomarking, Prevail Therapeutics, Quanterix, Rush University, Spear Bio, Syndeio Biosciences, Tymora Analytical Operations, and the University of Kentucky. J.L.D. has received research support from ADx Neurosciences, Eli Lilly and Company, Fujirebio, and Roche Diagnostics in the past 2 years. J.L.D. has received speaker fees from Eli Lilly and Company and LabCorp. J.L.D. is a founder and advisor for Dage Scientific and Monument Biosciences. J.L.D. has stock or stock options in ALZpath, Eli Lilly and Company, Genotix Biotechnologies, MindImmune Therapeutics, Monument Biosciences, and Neurogen Biomarking. H.C.G. holds the McMaster‐Sanofi Population Health Institute Chair in Diabetes Research and Care. H.C.G. reports research grants from AstraZeneca, Eli Lilly and Company, Merck, Novo Nordisk, and Sanofi; honoraria for speaking engagements from AstraZeneca, Boehringer Ingelheim, Eli Lilly and Company, Novo Nordisk, and Sanofi; and consulting fees from Abbott, AstraZeneca, Boehringer Ingelheim, Eli Lilly and Company, Janssen, Kowa, Merck, Novo Nordisk, and Sanofi. Author disclosures are available in the Supporting Information.

## CONSENT STATEMENT

All patients provided informed consent for participation in the study before any study‐specific procedures.

## Supporting information



Supporting Information

Supporting Information
