# Supplementary material for: Dulaglutide and neurodegeneration biomarkers: REWIND post hoc analysis
Source: Alzheimers Dement. 2026 Apr 16;22(4):e71391. doi: 10.1002/alz.71391 (PMC13084528; doi:10.1002/alz.71391)
Supplement: Supplementary file 1 — Supporting Information [file ALZ-22-e71391-s002.docx]

S**UPPLEMENTAL TABLE 1.** Baseline-adjusted LS means and percent change from baseline in biomarkers at 2 years by treatment.

| **Biomarker/subcategory** | **Treatment** | **LS mean** | **Change from**  **baseline, %** | ***P* value** |
| --- | --- | --- | --- | --- |
| **NfL** |  |  |  |  |
| Overall | Dulaglutide | 29.82 | 9.06 | .47 |
|  | Placebo | 30.13 | 10.19 |  |
| ≥56 pg/mL | Dulaglutide | 63.83 | −27.36 | .003 |
|  | Placebo | 75.40 | −14.20 |  |
| BL P-tau217 <0.25 pg/mL | Dulaglutide | 28.53 | 10.49 | .83 |
|  | Placebo | 28.43 | 10.10 |  |
| BL P-tau217 ≥0.25 pg/mL | Dulaglutide | 33.20 | 5.02 | .07 |
|  | Placebo | 34.88 | 10.33 |  |
| **P-tau217** |  |  |  |  |
| Overall | Dulaglutide | 0.21 | 5.77 | .29 |
|  | Placebo | 0.21 | 4.76 |  |
| <0.25 pg/mL | Dulaglutide | 0.18 | 12.64 | .43 |
|  | Placebo | 0.18 | 11.73 |  |
| ≥0.25 pg/mL | Dulaglutide | 0.31 | −9.23 | .46 |
|  | Placebo | 0.31 | −10.47 |  |
| **GFAP** |  |  |  |  |
| Overall | Dulaglutide | 160.28 | 8.91 | .07 |
|  | Placebo | 157.68 | 7.14 |  |
| <121 pg/mL | Dulaglutide | 101.37 | 17.06 | .31 |
|  | Placebo | 99.65 | 15.07 |  |
| ≥121 to ≤180 pg/mL | Dulaglutide | 160.53 | 9.00 | .09 |
|  | Placebo | 157.17 | 6.72 |  |
| >180 pg/mL | Dulaglutide | 253.43 | 1.18 | .52 |
|  | Placebo | 250.88 | 0.16 |  |

Abbreviations: BL, baseline; GFAP, glial fibrillary acidic protein; LS, least squares; NfL, neurofilament light chain; P-tau217, phosphorylated tau217.
